# Cancer‐Related Microangiopathic Haemolytic Anemia Revealing Occult Metastatic Gastrointestinal Signet‐Ring Cell Carcinoma: A Clinicopathological Case Report and Practical Differential Diagnostic Approach

**DOI:** 10.1002/ccr3.73675

**Published:** 2026-10-02

**Authors:** Danijela Jovanovic, Snezana Sretenovic, Dalibor Jovanovic, Milena Ilic, Predrag Djurdjevic

**Affiliations:** ^1^ Hematology Clinic University Clinical Center Kragujevac Kragujevac Serbia; ^2^ Faculty of Medical Sciences, Department of Internal Medicine University of Kragujevac Serbia; ^3^ Department of Pathology University Clinical Center Kragujevac Kragujevac Serbia; ^4^ Faculty of Medical Sciences, Department of Pathology University of Kragujevac Serbia

**Keywords:** bone marrow aspiration, cancer‐related microangiopathic hemolytic anemia (CR‐MAHA), gastrointestinal signet‐ring cell carcinoma, occult malignancy, trephine biopsy

## Abstract

This case highlights key laboratory red flags and a failure to respond to standard treatment, both of which should prompt urgent investigation for occult malignancy in patients presenting with cancer‐related microangiopathic hemolytic anemia (CR‐MAHA). Early recognition of these findings is crucial for timely diagnosis of the underlying malignancy.

## Key Clinical Message

Cancer‐related microangiopathic hemolytic anemia should be considered early in patients with non‐immune hemolytic anemia and thrombocytopenia, once other thrombotic microangiopathies have been carefully excluded, while it frequently coexists with disseminated intravascular coagulation (DIC). Early bone marrow examination may provide the first evidence of occult metastatic malignancy, thereby substantially shortening the diagnostic pathway.

## 1 Introduction

Microangiopathic hemolytic anemia (MAHA) is a clinicopathological syndrome characterized by Coombs‐negative hemolytic anemia and red cell fragments (schistocytes) in the peripheral blood, resulting from the mechanical destruction of erythrocytes within the microvasculature [1, 2]. Although thrombotic thrombocytopenic purpura (TTP) and hemolytic uraemic syndrome (HUS) are among the most common causes of MAHA, this hematological syndrome may also occur in association with disseminated malignancy, a condition referred to as cancer‐related microangiopathic hemolytic anemia (CR‐MAHA) [3].

Early recognition of CR‐MAHA is challenging but essential because it frequently mimics primary TMAs, particularly TTP and HUS, and shares several clinical and laboratory features with DIC, which may coexist with it. This distinction is particularly important because the diagnostic evaluation, management, and prognosis vary substantially among these conditions [3, 4, 5]. Cancer‐related MAHA represents one of the most challenging forms of secondary TMA, as it may be the initial manifestation of an underlying malignancy and precede its diagnosis [3, 6]. This case underscores the importance of recognizing hematological manifestations as potential early indicators of occult malignancy. In this patient, these findings prompted an expedited diagnostic work‐up, which ultimately led to the diagnosis of metastatic signet‐ring cell carcinoma of gastrointestinal origin. In addition to describing a rare presentation, this report outlines a practical approach to distinguishing CR‐MAHA from other thrombotic microangiopathies in routine clinical practice.

## 2 Case Report

A previously healthy 57‐year‐old woman was initially referred to her general practitioner in April 2026 with back pain radiating to the right leg. She received a five‐day course of intramuscular corticosteroids, a muscle relaxant, and diclofenac. Although this provided slight pain relief, extensive hematomas developed at the injection sites, prompting laboratory investigations. Later that day, her family took her to a private clinic, where she underwent magnetic resonance imaging (MRI) of the lumbosacral spine. Her initial complete blood count (CBC) revealed severe normocytic anemia, with a hemoglobin concentration of 66 g/L, and mild thrombocytopenia, with a platelet count of 107 × 10^9^/L. These findings were accompanied by elevated bilirubin levels; however, no other significant abnormalities were identified in the basic biochemical profile. She was referred immediately to the Centre for Urgent Medicine and subsequently admitted to the Hematology Clinic later that afternoon for further evaluation. The initial hematological work‐up, performed the following morning, demonstrated Coombs‐negative hemolytic anemia, with total and direct bilirubin concentrations of 37.8 and 13.0 μmol/L, respectively. Lactate dehydrogenase (LDH) was markedly elevated at 1744 U/L; schistocytes comprised 30% of the peripheral blood smear, the reticulocyte count was 14.2%, and haptoglobin was undetectable at 0.0 g/L. Her platelet count declined rapidly to 61 × 10^9^/L the next day. Additional biochemical investigations revealed an elevated alkaline phosphatase concentration of 596 U/L (reference range, 34–104 U/L), a uric acid concentration of 489 μmol/L, a creatine kinase concentration of 384 U/L, and mild abnormalities in the lipid profile. Initial coagulation studies revealed a fibrinogen concentration of 1.28 g/L, a prothrombin time (PT) of 17.4 s, an international normalized ratio (INR) of 1.35, an activated partial thromboplastin time (aPTT) of 27.8 s, and a D‐dimer concentration of > 20 μg/mL. Antithrombin activity was 112%, and the disseminated intravascular coagulation (DIC) score was 4. On the third day of hospitalization, her platelet count decreased further to 29 × 10^9^/L. The PLASMIC score was 6, indicating a high probability of thrombotic thrombocytopenic purpura (TTP). Blood samples were therefore obtained for ADAMTS13 activity testing, and plasma exchange was initiated alongside parenteral corticosteroid therapy. By the fourth day of hospitalization, there had been no improvement in either the patient's clinical condition or laboratory parameters. ADAMTS13 activity was subsequently found to be normal at 108.4%, effectively excluding TTP and supporting a diagnosis of secondary microangiopathic hemolytic anemia (MAHA). Plasma exchange and corticosteroid therapy were therefore discontinued. As the fibrinogen concentration had decreased further to 1.03 g/L, fresh frozen plasma replacement was initiated.

Over the subsequent three days, her platelet count remained below 40 × 10^9^/L. Further laboratory investigations revealed markedly elevated tumor‐marker concentrations, including carcinoembryonic antigen (CEA) at 101.7 μg/L and carbohydrate antigen 72–4 (CA 72–4) at > 500 U/mL (reference value, < 6.90 U/mL). The concentrations of CA 19–9, CA 125, and CA 15–3 were within their respective reference ranges. She was scheduled to undergo multislice computed tomography (MSCT) of the thorax, abdomen, and pelvis, together with mammography. Meanwhile, the previously performed spinal MRI was reviewed and interpreted as showing multiple secondary deposits involving all visualized vertebrae and the pelvic bones. MSCT of the thorax, abdomen, and pelvis, performed on the sixth day of hospitalization, revealed bilateral adrenal enlargement with imaging features suggestive of adenomas. Moderate abdominal lymphadenopathy was also identified, predominantly along the iliac vessels, with lymph nodes measuring up to 25 mm in diameter. Mammography revealed no abnormalities.

On the seventh day of hospitalization, revealed macroscopically normal gastric mucosa. Multiple gastric biopsies were obtained for histopathological evaluation and demonstrated preserved gastric architecture, with mild lymphocytic infiltration and discrete oedema, consistent with chronic antral gastritis. On the same day, bone marrow aspiration and trephine biopsy were performed to obtain prompt histopathological evidence of the suspected malignancy. The bone marrow aspirate revealed clusters of metastatic cells, confirming bone marrow involvement. The cells were exceptionally large, several times the size of a neutrophil, and contained eccentrically located nuclei occupying approximately three‐quarters of the cell volume, together with abundant pale cytoplasm (Figure 1). Bone marrow aspiration was the first diagnostic procedure to provide strong evidence of metastatic epithelial malignancy and substantially narrow the differential diagnosis before definitive histopathological confirmation. The trephine biopsy demonstrated near‐complete replacement of the normal hematopoietic tissue by metastatic adenocarcinoma. The tumor consisted predominantly of discohesive cells with abundant intracytoplasmic mucin, which displaced the nuclei peripherally, producing a characteristic signet‐ring‐cell morphology. Immunohistochemical analysis showed tumor‐cell expression of CK7, CK20, CDX2, MUC1, MUC2, and MUC5, whereas GATA3 was negative. This immunophenotypic profile, together with the characteristic morphology and markedly elevated CA 72–4 concentration, strongly supported a gastrointestinal primary and was most consistent with metastatic gastric signet‐ring‐cell adenocarcinoma. The absence of GATA3 expression made primary breast or urothelial carcinoma less likely (Figure 2). Colonoscopy was not performed because of the patient's poor performance status. She was subsequently referred to oncology; however, cancer‐directed treatment could not be initiated because of persistent severe thrombocytopenia. The patient died six days later.

**FIGURE 1:**
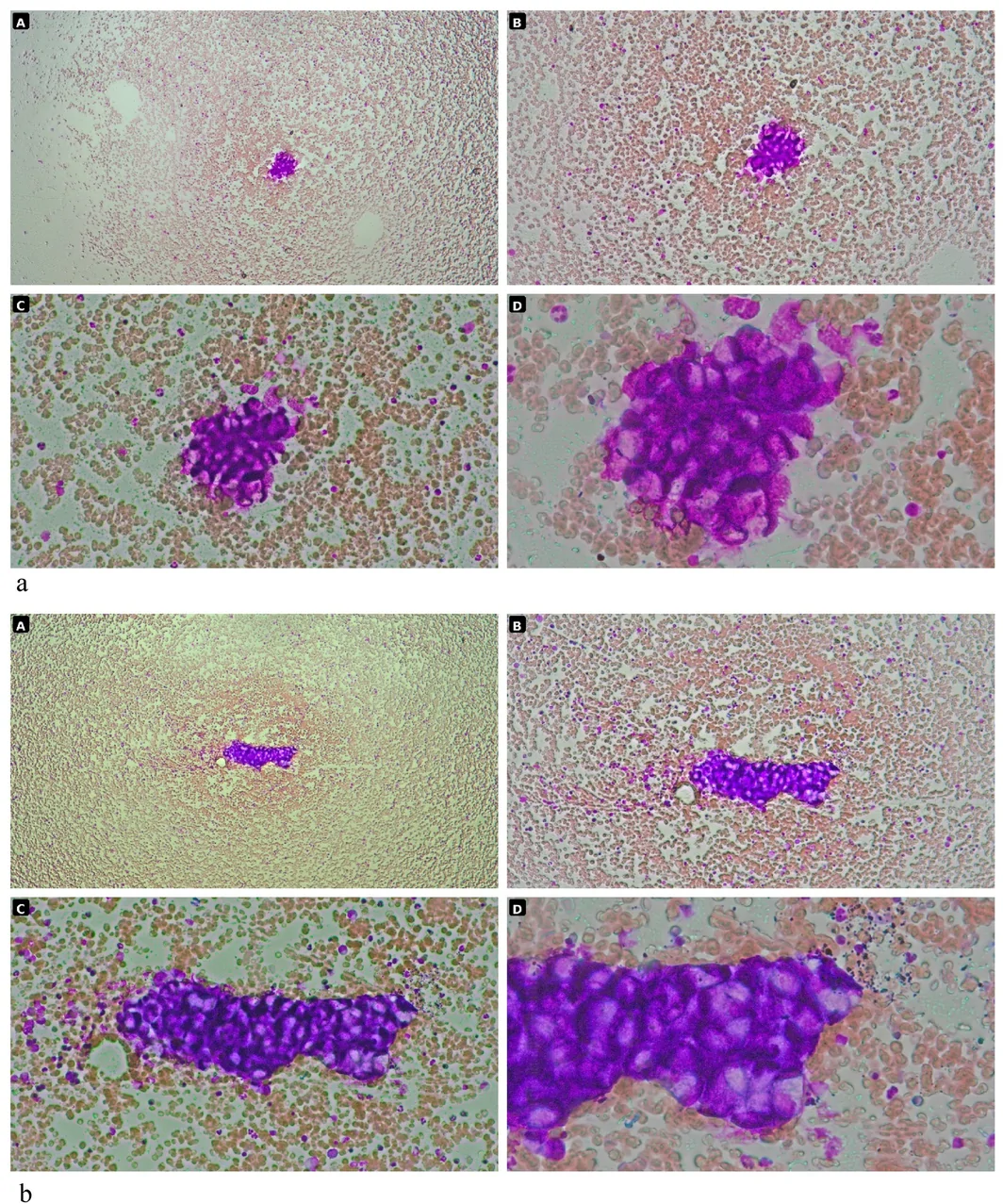
Bone marrow aspirate showing clusters of metastatic cells. (A) May–Grünwald–Giemsa (MGG) staining at ×50 magnification, showing an isolated cluster of non‐hematopoietic cells. (B) MGG staining at ×100 magnification, showing an isolated cluster of non‐hematopoietic cells. (C) MGG staining at ×200 magnification, showing a cluster of non‐hematopoietic cells with abundant pale cytoplasm and eccentrically positioned nuclei. (D) MGG staining at ×400 magnification, showing metastatic cells with characteristic morphology and markedly increased size compared with normal hematopoietic cells.

**FIGURE 2:**
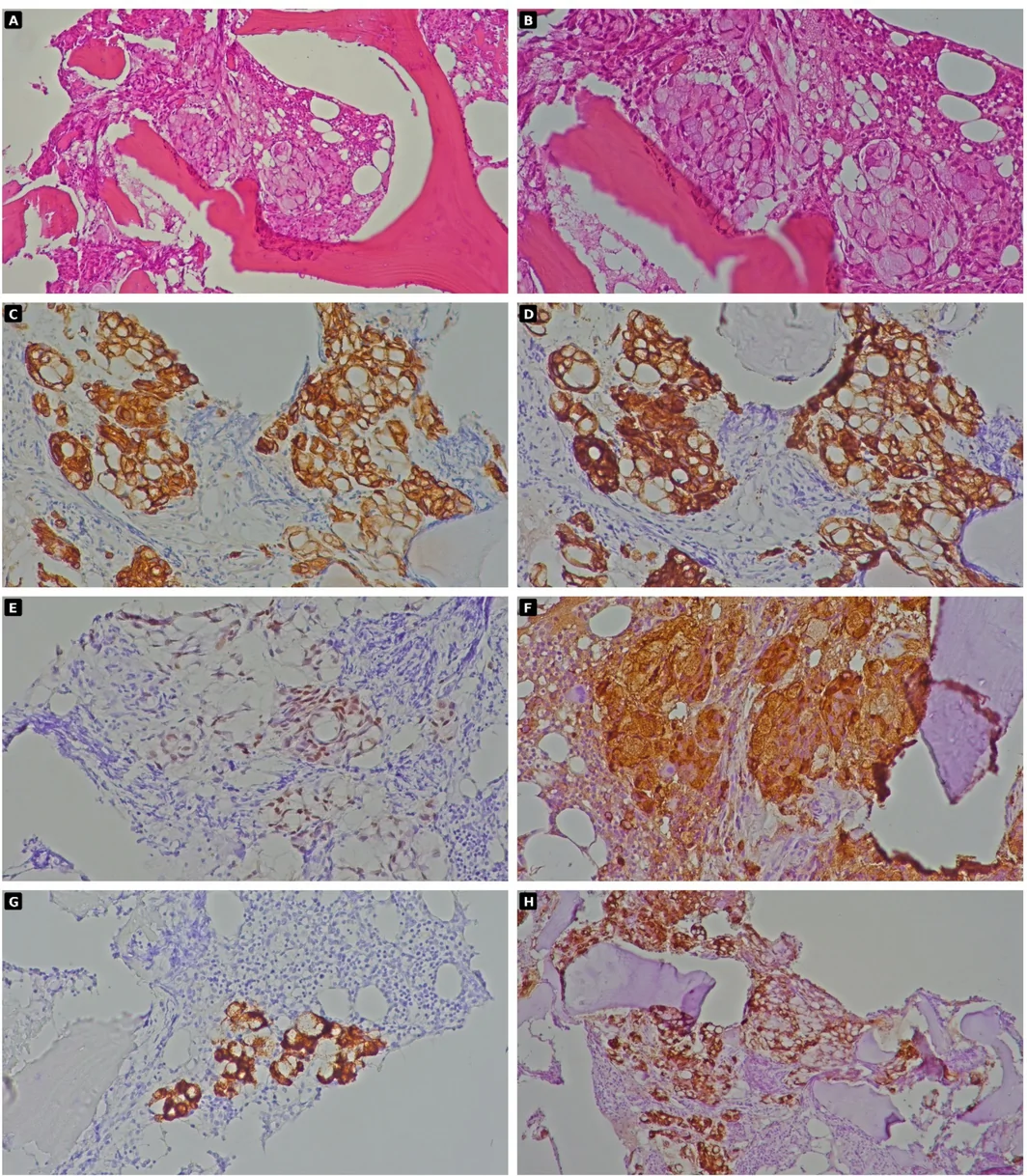
Bone marrow trephine biopsy showing the histological and immunohistochemical features of metastatic gastric signet‐ring‐cell carcinoma. (A) Hematoxylin and eosin (H&E) staining at ×50 magnification, showing extensive replacement of normal hematopoietic marrow by metastatic tumor infiltrates arranged in diffuse sheets and clusters. (B) H&E staining at ×100 magnification, showing discohesive tumor cells with abundant intracytoplasmic mucin displacing the nuclei peripherally and producing characteristic signet‐ring‐cell morphology. (C) CK7 immunohistochemistry at ×200 magnification, showing diffuse, strong cytoplasmic positivity in the tumor cells. (D) CK20 immunohistochemistry at ×200 magnification, showing positive cytoplasmic expression in the tumor cells. (E) CDX2 immunohistochemistry at ×200 magnification, showing nuclear positivity, supporting gastrointestinal differentiation. (F) MUC1 immunohistochemistry at ×200 magnification, showing strong cytoplasmic and membranous positivity, consistent with a mucin‐producing adenocarcinoma phenotype. (G) MUC2 immunohistochemistry at ×200 magnification, showing positive cytoplasmic staining, further supporting mucinous or intestinal differentiation. (H) MUC5 immunohistochemistry at ×200 magnification, showing strong cytoplasmic positivity and highlighting abundant mucin production by the metastatic tumor cells.

## 3 Discusion

Cancer‐related microangiopathic hemolytic anemia (CR‐MAHA) was first described by Brain et al. in 1970 and distinguished from other thrombotic microangiopathies (TMAs) by its association with an underlying malignancy [6]. The authors suggested that the syndrome was particularly associated with mucin‐producing malignancies, most commonly those originating in the stomach, breast, and lungs [6]. Since then, CR‐MAHA has remained rare and has been described mainly in individual case reports and small case series [4, 7, 8, 9, 10, 11, 12]. Brain et al. also proposed a potential pathophysiological mechanism involving tumor embolisation of the microvasculature and in situ thrombosis in the context of disseminated malignancy [6].

### 3.1 Epidemiology

The largest systematic review of cancer‐related microangiopathic hemolytic anemia (CR‐MAHA), comprising 168 reported cases, found that adenocarcinomas accounted for the vast majority of associated solid tumors [3]. Gastric carcinoma was the most frequently reported primary tumor (26.2%), followed by breast carcinoma (21.4%), prostate carcinoma (13.9%), and lung carcinoma (9.5%). Hematological malignancies accounted for 8.3% of cases, whereas 21% were classified as cancers of unknown primary origin. Importantly, more than 90% of patients had metastatic disease, and bone marrow infiltration was documented in more than 80% of cases in which marrow involvement was assessed [3]. These findings underscore the close association between disseminated malignancy, bone marrow involvement, and the development of CR‐MAHA. This pattern is consistent with that observed in our patient, who presented with extensive bone marrow infiltration and widespread metastatic disease at the time of diagnosis [3, 11, 12, 13, 14].

Mucin‐producing tumors appear to be particularly prominent among the associated adenocarcinomas, with signet‐ring‐cell morphology representing one of the most frequently reported histological patterns in this syndrome. Gastric signet‐ring‐cell carcinoma (SRCC) is an uncommon but increasingly recognized entity that reportedly accounts for a substantial and increasing proportion of gastric adenocarcinomas, particularly among younger patients and women [15]. Although its prognostic significance remains debated after stratification by stage [16], SRCC is generally associated with a diffuse growth pattern according to the Lauren classification [17], early peritoneal and bone marrow dissemination, and an aggressive clinical course [18]. These characteristics may partly explain its disproportionate association with CR‐MAHA.

### 3.2 Pathophysiology of CR‐MAHA

Several mechanisms have been proposed to explain the pathophysiology of cancer‐related microangiopathic hemolytic anemia (CR‐MAHA), and these may vary according to tumor type and biological characteristics. One of the most consistently recognized features of malignancies associated with CR‐MAHA is their capacity to produce mucin [19, 20]. Although mucins comprise a diverse group of glycoproteins, they share several biological properties that are relevant to tumor‐associated thrombosis [20]. Mucins can interact with P‐selectin on platelets and L‐selectin on leukocytes, thereby promoting the formation of platelet–leukocyte aggregates [21]. Clinical manifestations of this interaction may include migratory thrombophlebitis, also known as Trousseau syndrome, and CR‐MAHA [21, 22]. Both syndromes may therefore involve interactions among circulating tumor‐derived mucins, leukocyte L‐selectin, and platelet P‐selectin, potentially without concomitant thrombin generation [21]. Further support for a role of mucin in the pathogenesis of MAHA comes from Kovacic et al., who reported MAHA in a patient with non‐invasive mucinous cysts; the condition resolved completely following surgical treatment [4]. In addition to mucin‐mediated mechanisms, many malignancies associated with CR‐MAHA exhibit strong tissue‐factor expression. Tissue factor may promote the release of highly procoagulant microvesicles, resulting in thrombin generation and diffuse fibrin deposition, which may manifest clinically as disseminated intravascular coagulation (DIC) [23]. Together with the previously described mucin‐mediated mechanisms, these processes may contribute to microvascular narrowing and create a substrate for intravascular erythrocyte fragmentation. Another characteristic feature commonly associated with CR‐MAHA is metastatic disease, particularly extensive bone marrow involvement. Loss of E‐cadherin expression has been proposed as a mechanism facilitating early tumor dissemination [24, 25]. In our patient, the markedly elevated CA 72–4 and CEA levels, together with the confirmed signet‐ring‐cell morphology and immunohistochemical evidence of mucin production, supported a mucin‐producing tumor phenotype. These findings further support the proposed role of tumor‐derived mucins in the pathogenesis of CR‐MAHA. Although bone marrow infiltration is frequently associated with cancer‐related microangiopathic hemolytic anemia (CR‐MAHA), the precise mechanism linking marrow carcinomatosis to erythrocyte fragmentation remains incompletely understood [7, 24]. Early studies proposed that mechanical obstruction of the microvasculature by tumor emboli and fibrin deposits was responsible, whereas more recent evidence suggests that endothelial activation, tumor‐derived microparticles, and local coagulation activation may act synergistically [10]. Given the unique architecture of bone marrow sinusoids, which consist of thin‐walled and highly permeable microvessels, metastatic tumor infiltration may create a microenvironment that is particularly susceptible to erythrocyte fragmentation [8]. However, direct experimental evidence supporting this hypothesis remains limited. Our patient's findings closely reflect this proposed mechanism. Bone marrow aspiration and trephine biopsy demonstrated extensive replacement of the marrow by metastatic signet‐ring‐cell adenocarcinoma, while the peripheral blood smear showed marked schistocytosis, with schistocytes comprising 30% of the red‐cell population, accompanied by severe Coombs‐negative hemolytic anemia. Although causality cannot be established, the coexistence of extensive marrow infiltration and profound microangiopathic hemolysis supports the concept that marrow carcinomatosis may contribute substantially to erythrocyte fragmentation in CR‐MAHA [3, 8, 26]. The abundant mucin production demonstrated by immunohistochemistry in our case is also consistent with one of the proposed mechanisms underlying the pronounced prothrombotic phenotype of gastric signet‐ring‐cell carcinoma.

### 3.3 Laboratory Red Flags

From a laboratory perspective, patients with cancer‐related microangiopathic hemolytic anemia (CR‐MAHA) frequently present with thrombocytopenia, elevated lactate dehydrogenase (LDH) levels, schistocytosis, and evidence of Coombs‐negative hemolytic anemia. These findings may be accompanied by abnormalities suggestive of consumptive coagulopathy, including hypofibrinogenemia and elevated D‐dimer levels [5, 11]. The coexistence of CR‐MAHA and disseminated intravascular coagulation (DIC), usually in the form of non‐overt DIC, has also been described [8, 11]. Consequently, patients with CR‐MAHA may initially be misdiagnosed with thrombotic thrombocytopenic purpura (TTP) and, less commonly, hemolytic uraemic syndrome (HUS). In our patient, the rapid decline in platelet count, severe hemolysis, PLASMIC score of 6, and initial clinical suspicion of TTP prompted immediate plasma exchange before the ADAMTS13 activity result became available. The subsequent finding of normal ADAMTS13 activity, together with the absence of clinical or laboratory improvement following plasma exchange, redirected the diagnostic evaluation towards secondary MAHA associated with disseminated malignancy [5].

The markedly elevated CEA and CA 72–4 concentrations, extensive bone marrow involvement by metastatic disease, and subsequent immunohistochemical confirmation of metastatic gastrointestinal signet‐ring‐cell adenocarcinoma established the underlying diagnosis. This sequence highlights the importance of maintaining a high index of suspicion for occult malignancy when MAHA occurs in the absence of severe ADAMTS13 deficiency or fails to respond to plasma exchange [17]. Our case further emphasizes that the diagnostic work‐up should not stop once TTP has been excluded. After severe ADAMTS13 deficiency has been ruled out, prompt investigation for an underlying malignancy, including tumor‐marker assessment, cross‐sectional imaging, and bone marrow examination when leucoerythroblastosis or otherwise unexplained cytopenias are present, may substantially shorten the time to diagnosis [3]. In our patient, bone marrow aspiration provided the first direct evidence of metastatic carcinoma and significantly accelerated the diagnostic process before confirmation by trephine biopsy.

Table 1 summarizes the key clinical and laboratory features of CR‐MAHA, TTP, and DIC.

**TABLE 1:** Practical differential diagnosis of CR‐MAHA, TTP, and DIC in patients presenting with microangiopathic hemolytic anemia.

Feature	CR‐MAHA	TTP	DIC
Underlying trigger	Disseminated malignancy (often mucin‐producing adenocarcinoma)	Severe ADAMTS13 deficiency (autoimmune or congenital)	Systemic activation of coagulation (sepsis, trauma, malignancy, obstetric causes)
ADAMTS13 activity	Usually normal (> 30%), occasionally mildly reduced, or elevated	Severely reduced (typically < 10%)	Normal or mildly reduced
Platelet count	Low to moderately low	Severely low	Variable, often low
Fibrinogen	Usually normal (may decrease if concomitant DIC)	Usually normal	Low (consumed)
PT/INR	Usually normal (may decrease if concomitant DIC)	Normal	Prolonged
aPTT	Usually normal (may decrease if concomitant DIC)	Normal	Prolonged
D‐dimer	Elevated	Normal or mildly elevated	Markedly elevated
Schistocytes	Present	Present (prominent)	May be present, less prominent
LDH	Elevated	Markedly elevated	Normal/Elevated
Bone marrow involvement	Common in metastatic carcinoma	Absent	Variable (depends on underlying disease)
Response to plasma exchange	Poor/absent	Rapid and significant	Not indicated
Primary treatment	Treatment of underlying malignancy; supportive care	Plasma exchange, corticosteroids, caplacizumab	Treatment of underlying cause; blood product support

*Note:* Table adapted from data reported in references 1–3, 5 and 26.

Abbreviations: ADAMTS13, a disintegrin and metalloproteinase with thrombospondin Type 1 motif, member 13; aPTT, activated partial thromboplastin time; CR‐MAHA, cancer‐related microangiopathic hemolytic anemia; DIC, disseminated intravascular coagulation; INR, international normalized ratio; LDH, lactate dehydrogenase; PT, prothrombin time; TTP, thrombotic thrombocytopenic purpura.

### 3.4 Treatment

Although CR‐MAHA has been recognized as a distinct syndrome for more than five decades, effective disease‐specific treatment remains limited [3]. Plasma exchange is a cornerstone of treatment for TTP; however, it generally provides no sustained therapeutic benefit in CR‐MAHA when the underlying malignancy remains untreated [26]. Corticosteroids, anticoagulation, and fresh frozen plasma may be appropriate in selected clinical circumstances, depending on the underlying coagulopathy and the patient's clinical status. The available literature consistently emphasizes that early recognition of the underlying malignancy and prompt initiation of cancer‐directed therapy represent the most important therapeutic interventions [3, 4, 26].

## 4 Conclusion

Early and accurate recognition of CR‐MAHA is essential to guide the diagnostic work‐up and avoid delays in identifying the underlying malignancy. Once TTP has been excluded through appropriate laboratory evaluation and other primary TMAs, including HUS, have been reasonably considered, the diagnostic focus should promptly shift towards investigating an underlying malignancy. In addition to tumor‐marker assessment and appropriate cross‐sectional imaging, bone marrow aspiration and trephine biopsy should be considered early in patients with suspected CR‐MAHA, particularly when unexplained cytopenias, a leucoerythroblastic blood picture, or other findings suggest bone marrow involvement. These procedures may provide the first evidence of an otherwise occult metastatic malignancy and substantially accelerate diagnostic confirmation.

## Author Contributions

**Danijela Jovanovic:** conceptualization, investigation, writing – original draft, methodology, funding acquisition, validation, visualization, writing – review and editing, formal analysis, project administration, supervision, resources, data curation. **Snezana Sretenovic:** investigation, writing – review and editing, validation. **Dalibor Jovanovic:** conceptualization, funding acquisition, writing – original draft, writing – review and editing, methodology, validation, formal analysis, project administration, software, resources. **Milena Ilic:** investigation, writing – review and editing, methodology, validation, formal analysis. **Predrag Djurdjevic:** supervision, conceptualization, funding acquisition, writing – review and editing, project administration.

## Funding

The research was funded by the Ministry of Science, Technological Development and Innovation of the Republic of Serbia, contract number 451‐03‐34/2026‐03/200111.

## Consent

Written informed consent for the publication of this case report and the accompanying images was obtained from the patient's relatives.

## Conflicts of Interest

The authors declare no conflicts of interest.

## Data Availability

Since we present a clinical case data are a part of pateint medical history stored in our dosumentation. I am nor quite sure about what means the data availibility. We are glad to share this unique case within your journal, and once it gets DOI number as this is open access journal it will be widly available, which authors are hoping for.
